# *Chlamydia trachomatis* among Youth - Testing Behaviour and Incidence of Repeat Testing in Stockholm County, Sweden 2010-2012

**DOI:** 10.1371/journal.pone.0163597

**Published:** 2016-09-27

**Authors:** Anna Nielsen, Gaetano Marrone, Ayesha De Costa

**Affiliations:** 1 Department of Women's and Children's Health, Karolinska Institutet, Stockholm, Sweden; 2 Department of Public Health Sciences, Karolinska Institutet, Stockholm, Sweden; Xavier Bichat Medical School, INSERM-CNRS - Université Paris Diderot, FRANCE

## Abstract

**Background:**

Widespread testing and screening for genital *Chlamydia trachomatis* is often advocated as an important method to halt the epidemic. Sweden has long tradition of opportunistic screening services. Nevertheless infections rates have continued to rise over the past two decades, despite increased access to testing and treatment services.

**Methods:**

In this retrospective cohort study we describe the testing behavior for genital *Chlamydia trachomatis* among youth in Stockholm County, with a focus on repeated testing. Specifically we (a) study positivity rates among single and repeat testers, we (b) estimate the incidence of repeat testing and the rates of infection in repeat testing episodes, and we (c) estimate time to repeat testing and factors associated with repeat testing. All youth (aged ≥12 and <26) that tested for *Chlamydia trachomatis* in one of 33 Youth Health Clinics in Stockholm County between 1 January 2010 and 31 December 2012 were included in the study.

**Results:**

The cohort comprised a total of 65,951 individuals who did 119,699 tests during the study period. 42% of youth were repeat testers, the incidence of repeat testing was 35.0/100 person years. The overall baseline prevalence was 7.9%. Positivity rates of baseline tests among repeat testers were nearly twice as high among single testers of either sex. These were 17.1% and 9.8% among male repeat and single testers respectively. The corresponding rates for women were 9.4% and 4.3%. Positivity rates among repeat tests did not decline compared to the overall baseline positivity. Baseline test result and sex significantly influenced the occurrence of repeat testing.

**Conclusion:**

Among repeat testers we found high rates of *Chlamydia trachomatis* both at baseline and at repeat tests which suggests the possibility that this group might be continuing to engage in unsafe sexual practices. Given the extent of repeat testing and the high positivity rates on repeat testing, further research among this group is required to inquire into reasons for repeated testing.

## Introduction

*Chlamydia trachomatis (C*.*trachomatis)* has been identified as a major public health problem in Europe and worldwide.[1] In Sweden, young people aged 15–29 account for the vast majority of all infections.[2] In recent years, several countries have introduced national screening or opportunistic screening for *C*.*trachomatis* to halt the epidemic.[1,3,4] The Swedish opportunistic screening approach for *C*.*trachomatis* has been held up as an example of success in bringing down *C*.*trachomatis* infection rates which fell to a low level in the mid-90s when widespread testing was introduced.[5,6,7,8] However, despite the initial positive effect and ongoing efforts, including increasing the availability of testing services, rates of reported *C*.*trachomatis* cases in Sweden have steadily increased over the past two decades.[2] In Sweden sexual risk-taking, including multiple sexual partners has increased over the past years.[9,10,11] At the same time condom use is considered low, 4 out of 10 youth report that they never/seldom use condom with a temporary partner.[10] The epidemic of *C*.*trachomatis* was given priority by the Swedish National Board of Health and Welfare with the launching of the National Guidelines for Preventing Chlamydia in 2009.[12] Primary prevention, i.e. increased *C*.*trachomatis* awareness and condom use and was advocated, as well as testing for *C*.*trachomatis*. Laws and regulations govern prevention strategies on a national level and regional policies control the preventive work in each County.[13,14] Current recommendations include widespread and prompt *C*.*trachomatis* testing for youth and young adults in Stockholm County, Sweden.[15] As treatment failure seldom occurs, a test of cure and/or re-testing after testing positive for *C*.*trachomatis* is not routinely recommended.[15,16] While all positive cases of *C*.*trachomatis* are reported to the Public Health Agency of Sweden, information about who uses the testing services and frequency of repeat testing is poor. Yet anecdotal unpublished information from midwives in the Youth Health Clinics (YHC) suggested that a significant proportion of youth using the testing services tended to test repeatedly.

The aim of this study was to describe the testing behavior for genital *Chlamydia trachomatis* among youth in Stockholm County, with a focus on repeated testing. Specifically to (a) study positivity rates among single and repeat testers, (b) to estimate the incidence of repeat testing and the rates of infection on repeat testing episodes (c) estimate time to repeat testing and factors associated with repeat testing. This secondary data analysis, focusing on youth who test repeatedly, is the first report of a series of studies to explore why despite widespread access to screening, testing and treatment, genital *C*.*trachomatis* infection rates continue to rise in Stockholm.

## Methods

### Study Design and Setting

This retrospective cohort study was comprised of youth aged 12–25 who tested for *C*.*trachomatis* at the YHC in Stockholm County between 1 January 2010 and 31 December 2012. The YHC play an essential role in primary prevention; i.e. sexual education of schoolchildren on a group level; condom information and distribution; and also sexual education and risk assessment on an individual level. Counselling, testing, and treatment are free of charge. Attending the YHC is voluntary and a *C*.*trachomatis* test is done on request by the youth, either independently or on following advice from the staff. There are 33 YHC in Stockholm County, serving a population of approximately 258100 youth.[17] Tests for *C*.*trachomatis* on samples (vaginal swabs, and/or urine tests) received at these clinics are analyzed in one of three laboratories in Stockholm.

### Data collection

Records of all the young people that came for a *C*.*trachomatis* test at one of the YHC in Stockholm County and who had test samples sent for screening are maintained at the testing laboratories. Details of *C*.*trachomatis* tests were identified by using the designated invoice code for each YHC. After ethical approval from Stockholm Ethical Board (registration number 2013/1399-31/2) data files containing personal code number (unique personal identifier for each person resident in Sweden), date of test, test result, and the name of the YHC where the tests was performed were sent via certified mail from the laboratories to the to the Public Authority of Statistics. At the Public Authority of Statistics, information regarding the person’s; sex, age at each test, origin (Swedish or foreign), the educational level of the individual and his/her parents, and area of residency in Stockholm County was added. The data was anonymised by removing the personal identifying numbers of individuals and replacing each with unique serial numbers. Individuals were unidentifiable once the data reached the research team.

### Study Participants

All youth aged ≥12 and <26 who tested for *C*.*trachomatis* in one of the YHC in Stockholm County between 1 January 2010 to 31 December 2012 were included in the study. Variables in the data set are described below.

**Age:** categorised by age at first test in 5 groups; <15, 16–17, 18–19, 20–21, >22

**Ever positive:** individuals with ≥1 positive tests were classified as ever positive.

**Single tester:** defined as an individual who did only one test during the study period.

**Repeat tester:** defined as an individual who did more than one test during the study period i.e a repeat test as defined below

**Baseline test:** the baseline measurement for each individual defined by the date an individual entered the study (first test)

**Single test:** test performed by a single tester

**Repeat test:** test order ≥2

**Income:** Mean male income for the municipality where the youth was registered was taken from Statistical Yearbook of Sweden 2013 (Table 13.7) [18] and used as a socioeconomic marker. Income was categorised as high (>400000 SEK per year) middle (300000–400000 SEK per year) and low income (<300000 SEK per year). For those youth from communes outside Stockholm County the income was not calculated.

**Education:** Mother’s educational level was used as a socioeconomic marker and categorised as 9 year compulsory school, upper secondary school, and university.

**Origin:** was categorised as Sweden born and foreign born.

### Data Management and Analysis

Data was obtained from the Public Authority of Statistics in seven different excel files (one file per laboratory containing date of test and test result (n = 3), one file containing year of birth, sex, and origin (n = 1), one file per each study period year containing information regarding educational level and municipality where the youth was registered (n = 3)). The different datasets were linked in STATA by using the unique serial numbers. Tests within 6 weeks after a previous positive test were excluded for analysis of repeat testing to allow for tests of confirmation of cure (n = 558). Although this is not official policy it is likely that youth test again short after treatment to reassure themselves of an infections free status. We also scanned the data for duplicates (2 tests for the same person on the same day with the same results). Same-day duplicates samples with inconsistent test results were excluded from the analysis (n = 222). In cases of same-day duplicates with consistent test results, one of the test results were removed (n = 86).

Descriptive analysis and cross tabulations with chi-square tests were used to describe the characteristics of the testers divided by single and repeat testers. Details of the tests were described separately for males and females. Incidence of repeat testing per 100 person-years was estimated. Exposure time was defined as the time between the baseline tests until the date of the first repeat test or until the end of the study period (31^st^ December 2012) for individuals who did not have a repeat test recorded. Rates of repeat testing and time to repeat test were assessed using Kaplan-Meier curves stratified by baseline test result and gender. Log rank test were used to compare the Kaplan-Meier curves. Factors associated with re-testing were assessed in a Cox regression model. Covariates included sex, age, origin, baseline test result, socio-economic status (represented by mother’s education and mean male income). P<0.05 were considered significant. All analysis was performed using STATA/IC 12.1 (Stata Corp. College Station, Texas, USA).

## Results

### General Characteristics

In total 65,951 youth (71.6% women) between the age of 12 and 25 were included in the study (Table 1); 58.0%; (n = 38,271) of whom tested only once. The median age for female and male testers were 19 (IQR 18–21) and 20 years (IQR 18–21) respectively. The majority of individuals were Swedish born (90.7% in the cohort, compared to 84.4% of inhabitants in Stockholm County in the age group). While 8.2% of individuals had a positive first test, overall 13.4% (8811 individuals) tested positive at least once during the study period (66.6% women and 34.4% men). In total 22% of the youth did two tests, 9.9% did 3 tests and 10.1% performed 4 tests or more.

**Table 1:** Individual Characteristics of 65951 youth attending the YHC in Stockholm County 2010–2012.

Variables	Single tester		Repeat tester		All	
	N	%	N	%	N	%
Individuals (row%)	38271	58.0	27680	42.0	65951	100
Sex*						
*Male*	13213	34.7	5458	19.7	18671	28.4
*Female*	24825	65.3	22213	80.3	47038	71.6
Age at first test (median)	20 (IQR 18–22)		19 (IQR 17–20)		19 (IQR 18–21)	
Origin #						
*Foreign born*	3535	9.3	2043	7.4	5578	8.5
*Swedish born*	34503	90.7	25628	92.6	60131	91.5
Income/geographical ¤						
*Low*	5871	15.3	4237	15.3	10108	15.3
*Middle*	22475	58.7	17283	62.5	39758	60.3
*High*	5571	14.6	4768	17.3	10339	15.7
*Outside Stockholm*	4354	11.4	1363	4.9	5717	8.7
Mothers education ^						
*9 year compulsory*	4265	11.7	2952	11.0	7217	11.4
*Upper secondary*	16489	45.3	12835	47.9	29324	46.4
*University*	15640	43.0	11024	41.1	26664	42.2

*sex missing for 242 observations.

^#^ origin missing for 242 observations.

^¤^income/geographical area missing for 29 observations.

^mother education missing for 2746 observations.

As seen in Table 2, a total of 119,699 *C*.*trachomatis* tests at the YHC in Stockholm County between 1 of January 2010 and 31 of December 2012 done by the 65,951 individuals were included in the study. In total, 45% of all tests in the data set were repeat tests (83.7% by women and 16.3% by men). The overall proportion of positive tests during the study period was 7.9% (n = 9507 tests); 6.8% (n = 6306) in tests by females; and 11.6% (n = 3201) among tests performed by males (Table 2). The median number of repeat tests among individuals was 3 (range 2–18).

**Table 2:** Description of positivity rates among single and repeat tests performed at the YHC in Stockholm County 2010–2012.

Variables		Male		Female		All	
		N	%	N	%	N	%
**No of tests**		27493	100	92206	100	119699	
Baseline tests	Among Single testers	13213	48.1	24825	26.9	38038	31.8
	Among repeat testers	5451	19.8	22191	24.1	27642	23.1
Repeat tests (order ≥2)		8829	32.1	45190	49.0	54019	45.1
**Overall positivity**		3201	11.6	6306	6.8	9507	7.9
Baseline test positivity	Overall	2227	11.9	3147	6.7	5374	8.2
	Among single testers	1297	9.8	1064	4.3	2361	6.2
	Among repeat testers	930	17.1	2083	9.4	3013	10.9
Repeat test positivity		974	11.0	3159	7.0	4133	7.7

### Positivity of tests at baseline and of repeat tests

The overall baseline positivity of *C*.*trachomatis* infection was 8.2%; 11.9% (95% CI: 11.5–12.4%) in men, and 6.7% (95% CI: 5.5–6.9%) among women (Table 2). Male single testers had a baseline *C*.*trachomatis* positivity of 9.8% (95% CI 9.3–10.3%) and women 4.3% (95% CI 4.0–4.5%). In individuals testing more than once, *C*.*trachomatis* positivity at baseline was 17.1% in men (95% CI 16.1–18.1%) and 9.4% in women (95% CI 9.0–9.8%) (Table 2). The overall positivity rate of repeat tests was similar to the overall positivity of baseline tests; 7.7% overall, 11.0% (95% CI 10.4–11.7%) in males and 7.0% (95% CI 6.8–7.2%) in females. The overall positivity rate for the 2^nd^ tests was 8.0% and for the 3^rd^ tests 7.9% and thereafter the positivity rates declined marginally.

### Timing and incidence of repeat testing

**Fig 1:**
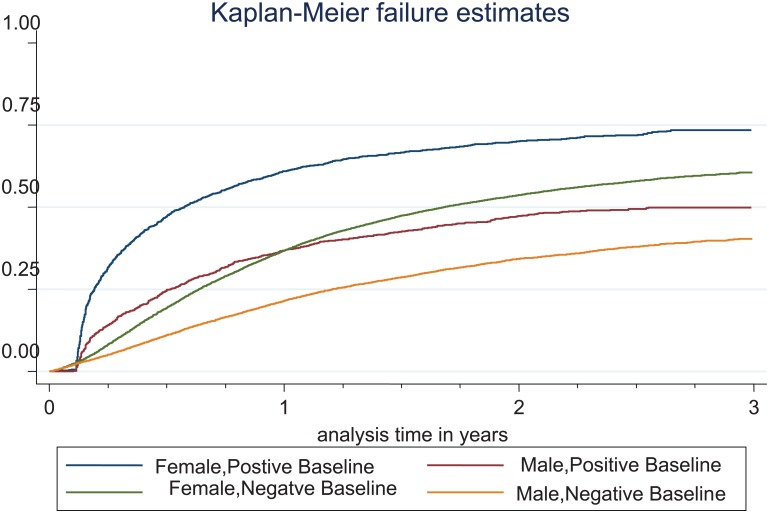
Kaplan-Meier failure estimate curves for incidence of repeat testing at the YHC Stockholm County stratified by sex and baseline status.

Among all repeat testers the median time between two tests were 168 days (IQR 83–312). For female repeat testers, the median values between were 167 days (IQR 83–307), and for male repeat testers the corresponding figures were 182 days (IQR 84–343). The follow-up time contributed by the 65,951 individuals was 78271 person-years. The incidence rate of repeat testing was 22.4/100 person-years among men (95% CI: 21.8–23.0) and 40.7/100 person years in women (95% CI: 40.1–41.2). Baseline status influenced both the re-testing frequency and time to the second test as shown in Fig 1. Men and women with a positive baseline test were more likely to re-test earlier than those negative at baseline. After 6 months approximately half of the women who tested positive at baseline and one quarter of men who tested positive at baseline had re-tested (Fig 1). Women positive at baseline had the highest incidence of re-testing, followed by women who tested negative at baseline. When stratifying by baseline status (positive/negative test result) and gender the incidence rate of repeat testing for female positive at baseline was 77.6/100 person-Years (95% CI: 74.1–81.1). For male testers positive at baseline the incidence rate of repeat testing was 35.4/100 person-years (95% CI: 33.1–37.8). Female testers, negative at baseline, had an incidence rate of repeat testing of 39.0/100 person-years (95% CI: 38.4–39.5). For men negative at baseline the corresponding figure was 20.9/ per 100 person-years (95% CI: 20.3–21.5). Log rank test showed significant differences in the rates and timing of repeat testing between male and females and between the different baseline status.(p<0.001).

### Factors associated with re-testing

The hazard of re-testing for *C*.*trachomatis* was significantly associated with age at first test, sex, income, education of mother and most strongly a positive baseline test (Table 3). Females were more likely to re-test compared to males (hazard ratio 1.8 p<0.01). Youth living in middle income areas and high income areas were more likely to re-test compared to youth living in low-income areas. Youth testing positive at baseline were more likely to re-test compared to those testing negative at baseline hazard ratio 1.99 p<0.01).

**Table 3:** Cox regression model, risk of re-testing for *C*.*trachomatis* among youth at the YHC in Stockholm County 2010–2012.

	Hazard Ratio	95% CI interval	P-value
**Age group**			
15 or less	Ref	ref	ref
16–17	0.98	0.92 1.04	0.43
18–19	0.95	0.89 1.00	0.07
20–21	0.76	0.72 0.81	**<0.01**
22 or more	0.39	0.37 0.42	**<0.01**
**Sex**			
Male	Ref	ref	ref
Female	1.80	1.74 1.85	**<0.01**
**Origin**			
Foreign born	Ref	ref	ref
Swedish born	1.05	0.99 1.10	0.05
**Mean male income for municipality**			
Low income	Ref	ref	ref
Middle income	1.06	1.02 1.09	**<0.01**
High income	1.17	1.12 1.22	**<0.01**
Outside Stockholm	0.68	0.63 0.72	**<0.01**
**Mothers educational level**			
Compulsory	Ref	ref	ref
Upper secondary	1.06	1.01 1.10	**<0.01**
University	1.00	0.96 1.04	0.92
**Baseline test result**			
Negative	Ref	ref	ref
Positive	1.99	1.91 2.07	**<0.01**

## Discussion

The main finding of this study was that repeat testing is common among youth using the services of the YHC in Stockholm County and that tests performed by repeat testers tend have high positivity rates. A little under half of all individuals (42%) tested more than once. In total 45% were repeat tests (≥2). The overall incidence of repeat testing was 22.4 per 100 person-years among men and 40.7 among women which are higher than previously reported from New Zealand (16.9 in men and 31.6 for women) and England (18.4 in men and 26.1 in women).[4,19] Although testing is advocated after engaging in risky sexual behaviour, there are studies that indicate that testing itself has little or even a negative effect on behavioral changes, i.e. adopting less risky sexual practices.[20,21] A phenomenon referred to as an “unintended screening effect” has been described in the literature, where a negative diagnosis of a sexually transmitted infection (STI) validates one’s behavior as not being overly risky and the importance of engaging in safer sexual practices is ignored.[22,23] Youth report perceiving themselves as “careful and responsible because they test frequently for *C*.*trachomatis*”, rather than seeing themselves as responsible because they adopt safer sexual practices. [10,24] Taken together with evidence of increased sexual risk taking and low condom use among the youth [9,10,11], it is possible that a proportion of young people use repeat *C*.*trachomatis* testing as a substitute to adopting less risky sexual practices. In the present study we found significantly higher baseline positivity rates in repeat testers which suggest that this group comprise those with sexual risk taking behaviour. Further repeat test positivity rates continue to stay as high as the overall baseline rates which indicates continued sexual risk taking after previous testing episode, i.e. repeat testing does not result in fewer infections on a group level. Although it is important in the short term to test and treat to prevent the spread of infection, the positivity rate of *C*.*trachomatis* is not decreasing over time. [2] Re-testing and treatment focus on the single current episode of infection and offer a temporary cure for the individual but possibly do not affect the behaviour of the individual in terms of practicing safer sex. A recent published Dutch study showed that *C*.*trachomatis* negatives were more likely to change into a more risky sexual behaviour compared to *C*.*trachomatis* positives who reported a more protective behaviour in terms of condom use.[25] Based on similar findings regarding the association between testing and sexual risk-taking [22,26] as well as results in the present study, it is likely that repeat testers comprise of youths with high risk behaviour, though this will need to be further explored. High rates of repeat testing and high rates of infections among repeat testers in our cohort imply that health care resources are being used inefficiently, i.e. a focus on testing and treatment services in the absence of adequate preventive work, will not result in a reduction of *C*.*trachomatis* infection rates if youth continue to engage in risky behaviours rather than adopt safer sexual practices.

Our cohort, as in other studies from youth settings,[27] had a high proportion of women. Only 28% of was male. Young women attend the YHC for numerous of reasons; contraceptive advise/prescription; gynaecological issues. It is likely to assume that young women also take the opportunity to test while at the clinic. Baseline tests done by males were twice as likely to be positive compared to tests done by females which could be explained by the fact that men are more likely to visit the clinic if they experience clinical symptoms of *C*.*trachomatis* infection.[28]

Baseline positivity predicted both the occurrence of, and the time to re-testing. Although men had higher rates of *C*.*trachomatis*, re-testing was more common among women. Women positive at baseline were more likely to re-test and do so in a shorter time interval. Similar results were presented from England where factors associated with testing more than once were being female and initially testing positive.[19,27]

### Limitations

An important limitation of this work is that we were not able to link our data, sourced from laboratory testing, with the clinical journal of the participant. This precludes the possibility of measuring sexual risk taking behaviour which might have been captured in the clinical records.

Many different health-clinics and STI-clinics (not particularly youth focused) in Stockholm offer STI-testing to the general population. In addition, home-based testing by on-line test requisition is increasingly popular. We chose to include only the YHC in this study as these clinics are mainly responsible for prevention of physical, psychological and sexual health among youth from a holistic point of view. The YHC offers opportunities for primary prevention and supporting sexual development of the youth. It is however reasonable to assume that the group who repeat tests for *C*.*trachomatis* at the YHC also test with home-based tests or at other STI-clinics or primary health clinics. Accordingly, the incidence of re-testing and the occurrence of *C*.*trachomatis* infection might be underestimated in the present study.

The time period we chose is a three year time interval. Youth in the cohort at either end of this time bracket might have tested at the YHC (before 1 January 2010 and after 31 December 2012) and therefore there may have been repeat testers who we classified as single testers. However this limitation only makes our estimates of repeat testing more conservative than they would otherwise be, and our arguments would still be valid.

### Strengths

The strength of this study is the large sample size among an age group that accounts for the majority of all *C*.*trachomatis* infections in Sweden and worldwide. By using unique serial numbers it was possible to retrospectively follow individuals over time.

## Conclusions

Our results indicate a high rate of repeat testing and relatively high rates of infections in repeat testers among the youth in Stockholm County. This together with evidence of increasing sexual risk taking and low condom use among youth [9,10,11] suggest the possibility of ongoing sexual risk taking despite previous tests and/or infection. Further research among the group of repeat testers will throw light on the motivations for repeat testing, and will help explore if the youth actually see repeated testing and treatment as a substitute or a preference to practicing safe sex. This will also have implications for a review of resources dedicated to preventive work with the youth and testing services.
